# Effect of Multicomponent Balance Training With Sensorimotor Foot Mobilization on Gait Adaptability and Functional Mobility in a Patient With Diabetic Peripheral Neuropathy: A Case Report

**DOI:** 10.7759/cureus.115823

**Published:** 2026-09-05

**Authors:** Rutuja Bhawad, Suraj Kanase

**Affiliations:** 1 Department of Neurosciences, Krishna College of Physiotherapy, Krishna Vishwa Vidyapeeth (Deemed to be University), Karad, IND

**Keywords:** diabetic peripheral neuropathy (dpn), dynamic gait index, gait adaptability, risk of fall, time up and go (tug)

## Abstract

Diabetic peripheral neuropathy (DPN) is a common complication of long-standing diabetes mellitus that leads to progressive sensory loss, proprioceptive deficits, and distal muscle weakness. These impairments collectively contribute to reduced balance, altered gait patterns, decreased functional mobility, and an increased risk of falls, ultimately affecting independence in daily activities. This case report presents the rehabilitation of a 58-year-old female diagnosed with DPN who reported bilateral plantar numbness, instability while walking, and difficulty performing routine functional tasks due to frequent imbalance. Following a comprehensive clinical assessment, an individualized physiotherapy program was designed with a focus on multicomponent balance training combined with sensorimotor foot mobilization. The intervention emphasized plantar sensory stimulation, postural control exercises under varying support conditions, task-specific gait retraining, and progressive functional mobility activities to enhance movement confidence and safety during ambulation. Two primary outcome measures were used to evaluate progress: the Dynamic Gait Index (DGI), assessing gait adaptability and dynamic balance during functional walking tasks, and the Timed Up and Go (TUG) test, used to determine functional mobility and fall risk. After six weeks of structured rehabilitation, the patient demonstrated improvement in both outcomes. The DGI score improved from 15/24 to 21/24, while the TUG time decreased from 16.8 seconds to 11.9 seconds, indicating improved gait adaptability and functional mobility. This case describes improvements following an early, individualized, and task-specific physiotherapy rehabilitation program in a patient with DPN.

## Introduction

Diabetic peripheral neuropathy (DPN) is one of the most common chronic complications of diabetes mellitus and is characterized by progressive damage to peripheral nerves, resulting in sensory, motor, and autonomic dysfunction. It significantly affects balance, gait, functional mobility, and overall quality of life, thereby increasing the risk of falls and disability among affected individuals [1]. The prevalence of DPN increases with the duration of diabetes and poor glycemic control, making it a major public health concern worldwide [2]. Sensory deficits, particularly involving proprioception and plantar sensation, contribute to impaired postural control and reduced functional independence in patients with DPN [3].

DPN is associated with a wide range of clinical manifestations, including numbness, tingling sensations, burning pain, muscle weakness, impaired balance, and gait abnormalities. These impairments often lead to decreased mobility, fear of falling, and limitations in activities of daily living. Furthermore, reduced physical activity resulting from mobility impairments may contribute to muscle weakness, decreased cardiovascular fitness, and a decline in overall functional capacity [4]. Early identification and management of these deficits are therefore essential to minimize disability and improve long-term outcomes. Physiotherapy plays a crucial role in addressing these impairments through targeted interventions aimed at improving gait and functional performance.

Physiotherapy is an essential component of multidisciplinary management in individuals with DPN, with a primary focus on improving balance control, gait performance, strength, and functional independence. Evidence supports the use of structured rehabilitation programs incorporating balance training, sensorimotor foot mobilization, strengthening exercises, gait training, and task-specific functional activities to improve postural stability and mobility outcomes [5,6]. Studies have also demonstrated that balance-focused interventions can significantly reduce fall risk in individuals with diabetes-related neuropathy [4-6]. In addition, altered postural control and instability patterns observed in DPN are well documented in the literature, further highlighting the need for targeted rehabilitation approaches [7,8].

Clinically, outcome measures such as the Dynamic Gait Index (DGI) and Timed Up and Go (TUG) test are widely used to assess dynamic balance, gait adaptability, functional mobility, and fall risk in individuals with peripheral neuropathy [6,9]. These tools provide objective insight into functional limitations and response to rehabilitation.

This case report describes the effects of a structured physiotherapeutic rehabilitation program combining multicomponent balance training and sensorimotor foot mobilization in a 58-year-old female patient with DPN. Although balance and gait training are commonly used in DPN, limited evidence is available regarding their combination with sensorimotor foot mobilization. This combined approach may provide complementary benefits by addressing both postural control and impaired plantar sensory input.

## Case presentation

Patient information

A 58-year-old female homemaker presented with symptoms such as numbness and tingling sensations in her feet, balance impairment, difficulty walking, and a fear of falling while performing daily household activities. The patient had been diagnosed with type 2 diabetes mellitus 12 years ago and had been experiencing symptoms of DPN for three years. She complained of an increasing problem with sensory function, with more prominence in the plantar surface of both her feet, which impeded her ability to walk safely. She had two episodes of falls during the last six months while walking on uneven surfaces.

The patient was capable of performing basic activities of daily living without assistance but had difficulties walking, negotiating stairs and prolonged walking due to poor balance, and decreased tolerance to walking. Clinically, she had diminished sensation in the plantar surface, poor proprioception at the ankle and toe joints, and mild weakness of the muscles responsible for postural instability and gait disturbances.

The patient had no prior medical history of stroke, Parkinson’s disease, vertigo, trauma, or any other condition that might be responsible for her problems in maintaining balance. She was recommended physiotherapy treatment to improve balance, functional mobility, and overall independence.

Clinical findings

On initial physiotherapy evaluation, the patient was conscious, oriented, and hemodynamically stable with normal vital signs. Examination was performed in both supported standing and functional positions as tolerated. Clinical examination revealed impaired balance, reduced postural stability, and difficulty in maintaining static and dynamic standing balance. Sensory examination demonstrated diminished plantar sensation bilaterally with impaired proprioception at the ankle and toes.

Functional assessment revealed limitations in functional mobility, particularly during community ambulation, stair negotiation, prolonged standing, and walking. The patient was independent in basic activities of daily living but experienced difficulty with complex and mobility-related tasks due to impaired balance and fear of falling. Muscle strength of the distal lower limb musculature was reduced, particularly in the ankle dorsiflexors and plantarflexors, contributing to gait instability.

Range of motion at the hip, knee, and ankle joints was within functional limits but associated with reduced control during movement due to impaired neuromuscular coordination. These deficits contributed to altered gait mechanics and reduced functional efficiency during mobility tasks.

Table 1 presents the chronological progression of the patient’s condition from long-standing type 2 diabetes mellitus to the development of DPN symptoms. The patient had experienced sensory symptoms and imbalance over the past few years, with increased fall risk in the six months before intervention. Baseline physiotherapy assessment (DGI and TUG) was performed on day one, followed by six weeks of structured rehabilitation. Post-intervention assessment was conducted at the end of treatment to evaluate functional improvements in balance and mobility.

**Table 1:** Chronological timeline of clinical events and physiotherapy rehabilitation in a patient with diabetic peripheral neuropathy. DGI: Dynamic Gait Index; TUG: Time Up and Go

Incident	Timeline
Diagnosis of type 2 diabetes mellitus	12 years prior
Onset of diabetic peripheral neuropathy symptoms (numbness, tingling in feet)	3 years prior
Episodes of imbalance and increased fall risk	6 months before intervention
Baseline physiotherapy assessment (DGI, TUG)	Day 1 of evaluation
Duration of rehabilitation	6 weeks
Post-intervention reassessment (DGI, TUG)	End of 6 weeks

Table 2 presents the manual muscle testing results of the bilateral lower limb muscle groups. The assessment demonstrated a reduction in muscle strength, more pronounced on the left side compared to the right. Hip and knee muscle groups showed near-functional strength on the right side, while the left side exhibited mild-to-moderate weakness.

**Table 2:** Manual muscle testing of lower limb musculature. Muscle strength was graded using the Medical Research Council (MRC) scale [10], where 0 = no visible contraction, 1 = trace contraction, 2 = active movement with gravity eliminated, 3 = active movement against gravity, 4 = active movement against moderate resistance, and 5 = normal muscle strength against full resistance. Grades with a plus sign (+) indicate strength slightly greater than the base grade but not sufficient to achieve the next full grade (e.g., 3+ = movement against gravity with minimal resistance).

Joint	Muscle group	Right side	Left side
Hip	Flexors	4	3+
	Extensors	4	3+
	Abductors	4	3
	Adductors	4	3
Knee	Flexors	4	3+
	Extensors	4	3+
Ankle	Plantarflexors	3+	3
	Dorsiflexors	3+	3

Distal musculature of the ankle, particularly the dorsiflexors and plantarflexors, showed comparatively greater weakness, indicating involvement of distal motor units commonly seen in DPN. This pattern of weakness contributed to impaired postural control, reduced gait efficiency, and difficulty in maintaining balance during functional activities.

Overall, the findings suggested that although gross antigravity strength was preserved in most proximal muscles, distal weakness significantly affected functional mobility and stability

Table 3 illustrates the deep tendon reflex and superficial reflex of the patient. Upper limb reflexes, including biceps, triceps, and supinator reflexes, were found to be normal bilaterally, indicating intact upper limb neurological function.

**Table 3:** Deep tendon reflex and superficial reflex examination. Reflex grading: ++ = normal, + = diminished, 0 = absent, ↓ = reduced response.

Reflex	Right side	Left side
Biceps	++	++
Triceps	++	++
Supinator	++	++
Knee (patellar)	+	+
Ankle (Achilles)	+	+
Plantar response	↓	↓

However, lower limb reflexes, particularly the patellar and Achilles tendon reflexes, were reduced bilaterally. The plantar response was also diminished, suggesting peripheral nerve involvement consistent with DPN.

The presence of reduced lower limb reflexes with preserved upper limb reflexes supported a length-dependent peripheral neuropathy pattern, which is characteristic of DPN. These neurological findings correlated with the patient’s clinical presentation of sensory loss, balance impairment, and functional mobility limitations.

Table 4 summarizes the clinical and neurological impairments observed in the patient with DPN. The patient exhibited reduced plantar sensation and impaired proprioception, which are key contributors to postural instability and balance dysfunction in DPN.

**Table 4:** Clinical and neurological findings in diabetic peripheral neuropathy.

Clinical parameter	Finding
Plantar sensation	Reduced bilaterally
Proprioception	Impaired at the ankle and toes
Distal muscle strength	Mild weakness in ankle dorsiflexors and plantarflexors
Balance status	Impaired static and dynamic balance
Gait pattern	Wide-based, cautious gait
Fall risk	Increased

Mild distal muscle weakness and altered gait pattern were also noted, reflecting neuromuscular impairment affecting lower limb control. The patient demonstrated an increased risk of falls due to compromised sensory feedback and reduced postural control mechanisms. Overall, these findings were consistent with the typical presentation of length-dependent peripheral neuropathy and explained the functional limitations observed during mobility tasks.

Clinical interpretation

The overall presentation was consistent with chronic DPN, characterized by distal symmetrical sensory loss, proprioceptive impairment, mild distal muscle weakness, and balance dysfunction. These impairments collectively contributed to reduced postural stability, altered gait mechanics, and increased risk of falls, thereby limiting functional independence.

Diagnostic assessment

The diagnosis of DPN was established based on clinical evaluation, neurological examination, and functional outcome measures. The patient presented with characteristic features of peripheral nerve involvement, including reduced plantar sensation, impaired proprioception, distal lower limb weakness, and balance dysfunction consistent with a length-dependent neuropathy pattern.

A detailed neurological assessment using the Michigan Neuropathy Screening Instrument (MNSI), which is a standardized and widely used tool for assessing DPN [11,12], further supported the diagnosis. The MNSI questionnaire score was 9/13, indicating the presence of peripheral neuropathy, with sensory impairment and clinical signs consistent with diabetic nerve involvement. Nerve conduction studies were not performed.

Functional assessment was focused on gait performance, balance-related mobility, and fall risk. The DGI was used to evaluate gait adaptability under varying task demands, including changes in speed, head movements, and obstacle negotiation [13]. The TUG test was used as a measure of functional mobility and fall risk [14]. Baseline assessment using DGI and TUG revealed impaired gait adaptability and increased fall risk, indicating reduced functional mobility.

Based on the integration of clinical findings, MNSI results, and functional outcome measures (DGI and TUG), a diagnosis of DPN with significant gait adaptability deficits and increased fall risk was established. These findings guided the formulation of an individualized physiotherapeutic rehabilitation program aimed at improving dynamic gait performance, enhancing functional mobility, and reducing fall risk through targeted multicomponent intervention.

Physiotherapy rehabilitation protocol

The rehabilitation program was designed as a structured, progressive physiotherapy protocol focusing on improving dynamic gait adaptability, postural control, and functional mobility, while simultaneously addressing fall risk associated with DPN. The intervention was individualized according to the patient’s baseline impairments, tolerance level, and weekly clinical response.

Progress throughout rehabilitation was monitored using the DGI to assess gait adaptability under varying functional conditions and the TUG test to evaluate functional mobility and fall risk. Treatment progression was guided by improvements in these outcome measures along with clinical observation of balance control and gait stability. Table 5 shows the detailed rehabilitation program.

**Table 5:** Physiotherapy rehabilitation protocol. DPN: diabetic peripheral neuropathy; ROM: range of motion; DGI: Dynamic Gait Index

Week	Goal	Therapeutic interventions
Week 1	Sensory activation, safety awareness, and initiation of postural control	Patient education regarding DPN, fall prevention, and safe mobility strategies. Plantar sensory stimulation using textured surfaces, cotton, and graded tactile inputs. Gentle ankle joint mobilization and active-assisted ROM exercises for lower limbs. Supported sitting and standing balance training. Weight-bearing exercises in standing with support. Breathing and relaxation exercises to improve tolerance
Week 2	Improve postural stability and initiate dynamic balance control	Progression to static and dynamic standing balance with reduced support. Multidirectional weight shifting exercises. Early gait initiation with supervision focusing on step placement and heel contact. Basic lower limb strengthening exercises emphasizing ankle stability and control
Week 3	Enhance gait adaptability and functional mobility	Dynamic gait training with changes in speed, direction, and head movements (DGI-based components). Obstacle negotiation, tandem walking, and step-over tasks. Functional closed-chain strengthening exercises. Supervised community-simulated walking activities to improve confidence and control
Week 4–6	Improve functional independence and reduce fall risk	Independent gait training with focus on smooth transitions, turning, and dual-task activities. Stair training and uneven surface walking practice. Endurance-based walking exercises. Functional mobility training for daily activities. Reinforcement of fall prevention strategies and progression of the home exercise program

Rehabilitation Protocol Overview (FITT Principles)

Frequency: Five days per week for six weeks.

Intensity: Gradually progressed based on patient response and safety.

Time: 40-60 minutes per session.

Type: Multicomponent balance training, sensorimotor foot mobilization, gait training, and functional task-oriented exercises.

Physiotherapy rehabilitation was initiated after the baseline assessment. Progression through each phase was guided by the patient’s clinical response, particularly improvements in postural stability, gait performance, and functional mobility, as reflected by changes in DGI and TUG scores.

Figure 1 shows a patient performing a single-leg balance exercise on an unstable balance disc while using the parallel bar for upper-limb support during physiotherapy rehabilitation.

**Figure 1:**
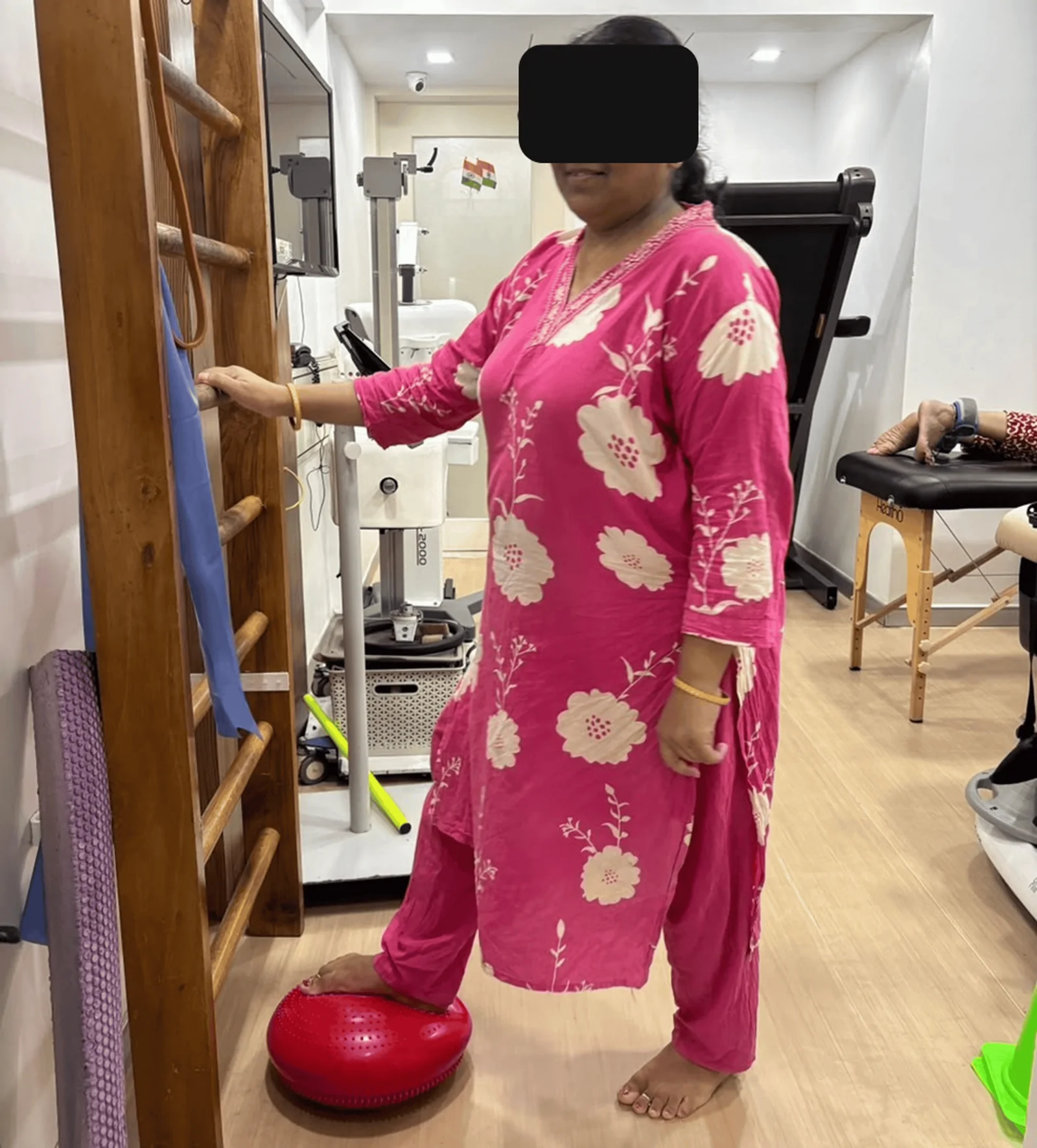
Patient performing single-leg balance exercise using a balance disc with upper-limb support.

Outcome progress

Table 6 presents the changes in functional outcomes following the rehabilitation program. Serial assessment using the DGI and TUG demonstrated progressive improvement in gait adaptability, functional mobility, and fall risk reduction over the intervention period. The observed changes reflect enhanced dynamic balance control and improved efficiency in functional ambulation following structured physiotherapy rehabilitation. Both outcomes demonstrated clinically significant improvement in gait adaptability and functional mobility following intervention.

**Table 6:** Changes in functional outcomes following the rehabilitation program.

Outcome measure	Pre-treatment	Post-treatment
Dynamic Gait Index	15/24	21/24
Timed Up and Go	16.8 seconds	11.9 seconds

## Discussion

DPN is a progressive complication of diabetes mellitus characterized by distal symmetrical sensory loss, proprioceptive impairment, and motor weakness, which collectively contribute to impaired postural control, gait dysfunction, and increased risk of falls [1,2]. In the present case, the patient demonstrated classical features of DPN, including reduced plantar sensation, impaired proprioception, and distal lower limb weakness, which are consistent with previously reported clinical patterns of diabetic neuropathy [3].

Following rehabilitation, improvement was observed in both DGI and TUG scores, indicating better gait adaptability and reduced fall risk. The improvement in DGI reflects better performance in complex walking tasks such as speed changes, head movements, and obstacle negotiation, suggesting improved dynamic postural control and functional gait efficiency. Similarly, the reduction in TUG time indicates improved functional mobility, sit-to-stand performance, and overall reduction in fall risk.

The observed functional gains may be attributed to the effects of multicomponent balance training combined with sensorimotor foot mobilization. Balance training is known to enhance sensory integration by improving the interaction between visual, vestibular, and somatosensory systems, which is often impaired in individuals with peripheral neuropathy [5,6]. Sensorimotor foot mobilization is likely to increase plantar afferent input, which may improve postural awareness and corrective responses during standing and gait activities [7].

Task-oriented and progressive gait training may have further facilitated motor learning and improved gait adaptability. Repeated exposure to functional walking tasks under varying conditions can promote more efficient motor strategies, leading to better stability during ambulation and reduced compensatory movement patterns [8]. In addition, functional task practice may have contributed to improved confidence during ambulation and reduced fear of falling, which is an important factor influencing mobility in individuals with DPN [9].

A key strength of this case report is the use of a structured, progression-based rehabilitation program with objective monitoring through DGI and TUG, allowing clear documentation of functional improvement in gait adaptability and fall risk over a six-week period. Unlike broader group-based studies, this case provides a focused view of individual response to rehabilitation, highlighting clinically significant changes in functional mobility within a relatively short duration.

However, as this is a single-case report, the findings cannot be generalized to all individuals with DPN. The lack of long-term follow-up also limits understanding of the sustainability of these improvements.

Limitations and future studies

This case report is limited by its single-patient design, which restricts the generalizability of the findings to a wider DPN population. Nerve conduction velocity studies were not performed, which is acknowledged as a limitation of the present case, as electrodiagnostic testing could have provided objective confirmation and characterization of peripheral nerve involvement. The lack of detailed exercise-level documentation, including specific repetitions, sets, duration, and standardized progression criteria for individual interventions, may limit the reproducibility of the rehabilitation protocol. In addition, long-term follow-up was not conducted to evaluate the sustainability of the functional gains achieved after the intervention period.

Future studies involving larger sample sizes, comparative study designs, and longer follow-up periods are recommended to establish stronger evidence for the effectiveness of multicomponent balance training with sensorimotor foot mobilization in individuals with DPN. Additionally, incorporation of objective gait analysis and advanced balance assessment tools may further strengthen future research outcomes.

## Conclusions

This case report demonstrates that a structured physiotherapy rehabilitation program incorporating multicomponent balance training combined with sensorimotor foot mobilization can produce significant improvements in gait adaptability and functional mobility in a patient with DPN. The observed improvement in DGI scores reflects enhanced dynamic balance control, better adaptability during complex gait tasks, and improved postural stability. Similarly, the reduction in TUG time indicates improved functional mobility, greater efficiency in transitional movements, and a reduced risk of falls. The findings suggest that early, individualized, and task-oriented physiotherapeutic intervention may play a crucial role in addressing balance and gait impairments associated with DPN. The integration of sensory re-education with progressive balance and gait training appears to be particularly effective in improving functional outcomes and enhancing independence in daily activities.
